# Clonal population expansion in an outbreak of *Plasmodium falciparum* on the northwest coast of Ecuador

**DOI:** 10.1186/s12936-015-1019-2

**Published:** 2015-12-10

**Authors:** Fabián E. Sáenz, Lindsay C. Morton, Sheila Akinyi Okoth, Gabriela Valenzuela, Claudia A. Vera-Arias, Eileen Vélez-Álvarez, Naomi W. Lucchi, L. Enrique Castro, Venkatachalam Udhayakumar

**Affiliations:** Centro de Investigación en Enfermedades Infecciosas y Crónicas, Escuela de Ciencias Biológicas, Pontificia Universidad Católica del Ecuador, Ave. 12 de Octubre 1076 y Roca, Quito, Ecuador; Malaria Branch, Division of Parasitic Diseases and Malaria, Centers for Global Health, Centers for Disease Control and Prevention, Atlanta, GA USA; Atlanta Research and Education Foundation, Decatur, GA USA; Ministerio de Salud Pública, Guayaquil, Ecuador; Universidad de las Fuerzas Armadas, Quito, Ecuador

**Keywords:** *Plasmodium falciparum*, Malaria, Ecuador, Outbreak, Microsatellite markers

## Abstract

**Background:**

Determining the source of malaria outbreaks in Ecuador and identifying remaining transmission foci will help in malaria elimination efforts. In this study, the genetic signatures of *Plasmodium falciparum* isolates, obtained from an outbreak that occurred in northwest Ecuador from 2012 to 2013, were characterized.

**Methods:**

Molecular investigation of the outbreak was performed using neutral microsatellites, drug resistance markers and *pfhrp2* and *pfhrp3* genotyping.

**Results:**

A majority of parasite isolates (31/32) from this outbreak were of a single clonal type that matched a clonal lineage previously described on the northern coast of Peru and a historical isolate from Ecuador. All but one isolate carried a chloroquine-resistant *pfcrt* genotype and sulfadoxine- and pyrimethamine-sensitive *pfdhps* and *pfdhfr* genotypes. *Pfmdr1* mutations were identified in codons 184 and 1042. In addition, most samples (97 %) showed presence of *pfhrp2* gene.

**Conclusions:**

This study indicates that parasites from a single clonal lineage largely contributed to this outbreak and this lineage was found to be genetically related to a lineage previously reported in the Peruvian coast and historical Ecuadorian parasites.

**Electronic supplementary material:**

The online version of this article (doi:10.1186/s12936-015-1019-2) contains supplementary material, which is available to authorized users.

## Background

Some countries in Latin America have achieved and surpassed the goals set by the World Health Assembly for reducing malaria cases (reduction of malaria burden by 75 % between 2000 and 2015) [1]. In particular, Ecuador has decreased the number of malaria cases from more than 100,000 (approximately 13.6 cases per 1000 individuals) in 2000 to 378 cases in 2013 (approximately 0.05 cases per 1000 individuals), thus placing it in the pre-elimination phase [1–3]. Nevertheless, outbreaks of both falciparum and vivax malaria still occur in both the Amazon and Coastal regions of Ecuador.

Molecular tools are valuable in providing insight about the potential source of malaria parasites in outbreak investigations [4–8]. Neutral microsatellite markers, single nucleotide polymorphism (SNP) based molecular barcodes and genomics tools have been used to characterize *Plasmodium falciparum* parasites in South America [5, 9–17]. These studies have suggested that *P. falciparum* parasites in this region have undergone population bottlenecks and in many parts of Peru and Colombia there are clonal population of parasites that are highly prevalent in different geographical regions.

One molecular investigation of *P. falciparum* isolates from Peru, using seven neutral microsatellite markers, revealed that the parasite isolates collected between 1999 and 2000 (during peak malaria expansion after the malaria elimination era) comprised five distinct clonal lineages named A, B, C, D, and E [12]. In the coastal region of Peru, all tested parasites belonged to the E clonal lineage while in the western region of the Peruvian Amazon, the C and D clonal lineages appeared more frequently; in the eastern and central Amazon A, B, C and D clonal lineages were found [12]. It was proposed that C, D and E clonal lineages may have originated in Ecuador and Colombia while A and B clonal lineages may have originated in Brazil [11]. The profile of drug resistance markers among these clonal lineages also varied and, by combining drug resistance marker profiles and microsatellite allele information, it was possible to further understand the genetic relationship among these parasite lineages [7, 8, 12, 13, 17]. Recent studies suggest that Peru has the highest known proportion of *pfhrp2* deleted *P. falciparum* parasites in the region, followed by Colombia and Suriname [14–16, 18]. Thus, by combining genetic markers such as neutral microsatellite markers, drug resistance markers and *pfhrp2* gene presence or absence one might identify parasite lineages originating in this region as demonstrated in recent outbreak investigations in Peru [7, 8].

**Fig. 1:**
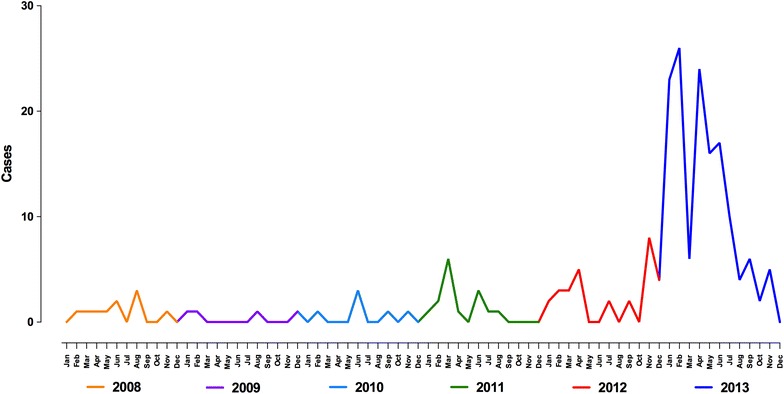
Number of reported cases of *Plasmodium falciparum* per month in Esmeraldas city from 2008 to 2013 (data: Statistics Department, SNEM, Ministry of Public Health). An increase in the overall reported number of cases can be seen at the end of 2012 and during 2013

From November 2012 to November 2013, an outbreak of *P. falciparum* occurred in the city of Esmeraldas in the northwest of the country. One-hundred and fifty-one cases of *P. falciparum* were reported during this period in sharp contrast to previous years where no more than ten cases were reported annually (Fig. 1). Understanding the origins of malaria cases will be useful in developing strategies to prevent future outbreaks.

In order to understand the source of this outbreak parasite population the following questions were answered: (a) are these parasite populations genetically similar to parasite populations in Ecuador, Peru (southern border) and Colombia (northern border)? (b) is there any evidence to suggest these parasites may have origins outside this region (Andean)? and (c) did these outbreak parasites belong to a single clonal lineage or to multiple lineages?

## Methods

### Ethics statement

The parasite samples used in this study were obtained from the malaria surveillance protocol approved by the Ethical Review Committee of Pontificia Universidad Católica del Ecuador. Written informed consent was provided by study participants and/or their legal guardians.

### Study site

Esmeraldas city is the largest city in Esmeraldas Province in northwest Ecuador. All samples used in this study were collected in Esmeraldas city during a malaria outbreak that occurred between November 2012 and November 2013.

### Samples

**Fig. 2:**
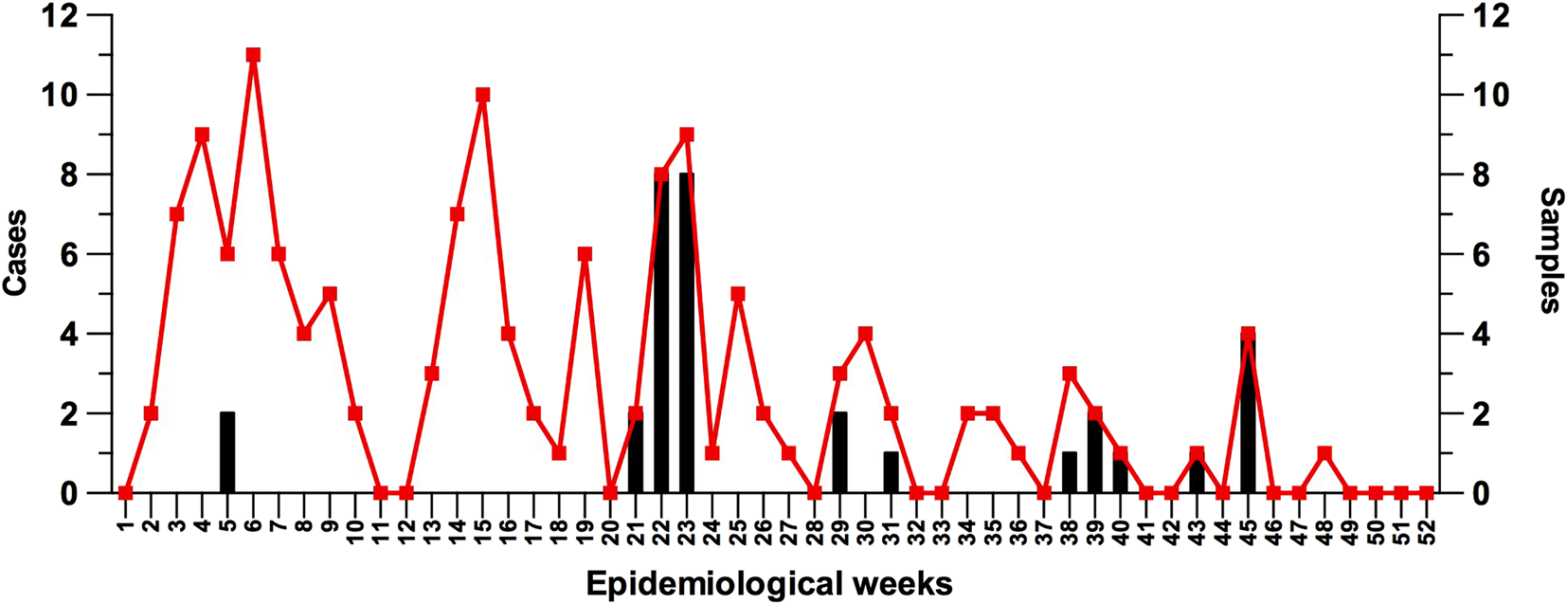
Number of samples collected during the outbreak. The *curve* shows the total number of cases reported in each epidemiological week during 2013. The *bars* correspond to the number of samples collected in different epidemiological weeks that were used in this study. The samples available for molecular study were collected only at selected time points as indicated

**Fig. 3:**
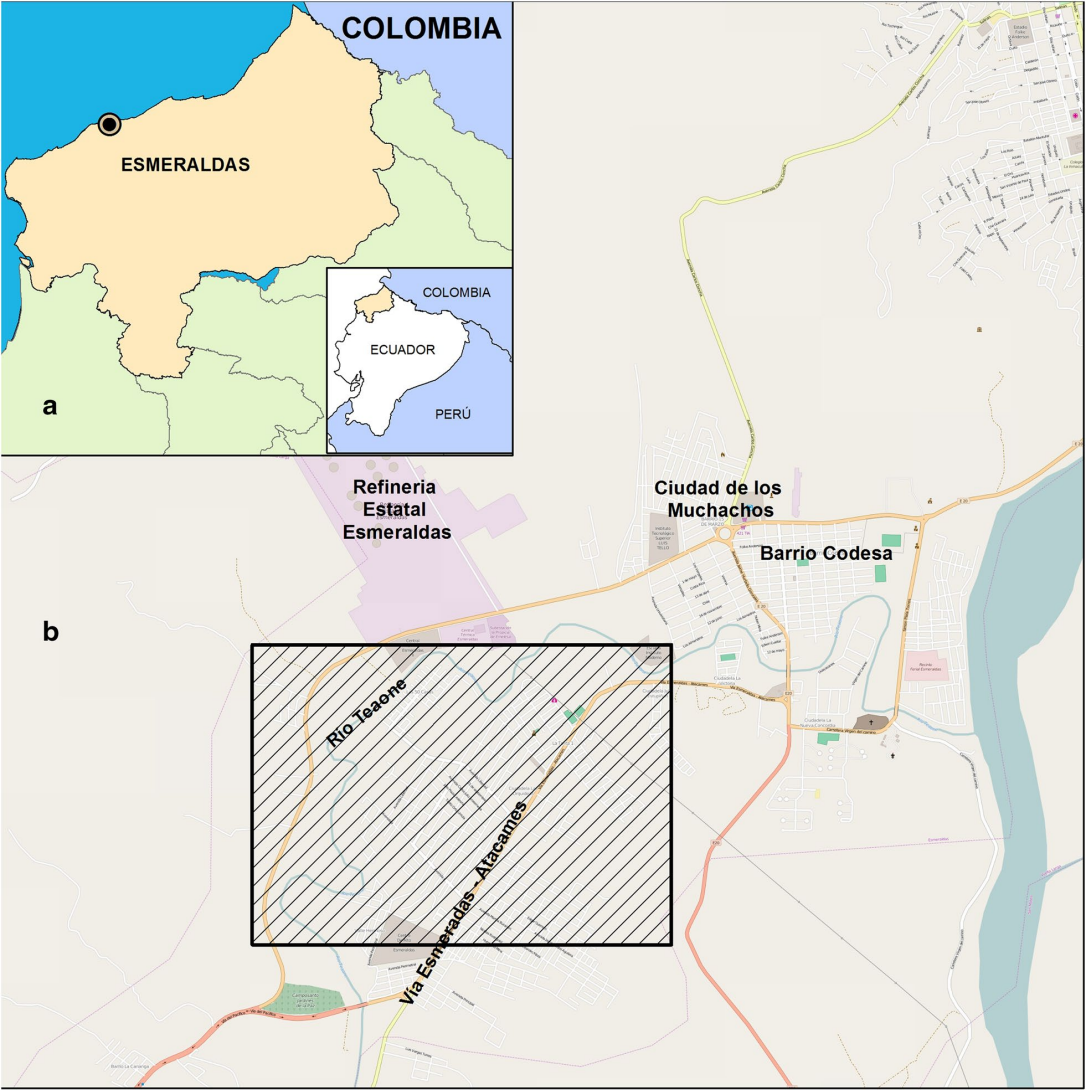
Study area. **a** Esmeraldas Province and its location in Ecuador. Esmeraldas city is marked as a *point* in the coast of the province. **b** Map of Esmeraldas city. Most (28; 88 %) of the patient samples were collected from the neighbourhoods in the south of the city (*black rectangle*) which corresponds to the area where most cases occurred

The malaria outbreak occurred between November 2012 and November 2013. Samples used in this study were collected from patients at 11 time points in 2013 between epidemiological weeks 5 and 45, with a higher concentration of samples collected in weeks 21–23 (Fig. 2). Differences in sample collection patterns were due to logistical challenges, including lack of informed consent, lack of sample collection and delayed training of microscopists. A total of 32 blood samples collected from patients who were initially reported to be microscopically positive for *P. falciparum* infection, and from whom informed consent was obtained, were available for this molecular investigation. The blood samples were collected by finger prick or by drawing peripheral whole blood and spotted on 3MM Whatman filter paper. All the samples were collected in Esmeraldas city between January and November 2013 and most (28 samples, 87.5 %) came from the neighbourhoods in the southern part of the city where the majority of cases were reported (Fig. 3).

### Reference isolates

In this study, laboratory adapted *P. falciparum* isolates 3D7, Dd2 and HB3 were used as controls for genotyping. In addition, Ecu 1110, a *P. falciparum* culture-adapted strain that was originally isolated in Ecuador in 1990 (provided by Dr Tom Wellems, National Institutes of Health, Bethesda, USA) was included as a reference strain from Ecuador.

### DNA extraction and confirmation of infection

DNA was isolated from filter paper or whole blood using the QIAamp DNA mini-spin kit (QIAGEN, Valencia, CA, USA) or Chelex-100 [19]. *Plasmodium falciparum* infection status was further confirmed by two different molecular methods: nested PCR using primers for 18S ribosomal RNA [20], and photo-induced electron transfer (PET) real-time PCR using specific fluorogenic primers for *Plasmodium* (genus) and *falciparum* (species) [21].

### Detection of the *pfhrp2* and *pfhrp3* genes by PCR

Nested PCR amplifications of *pfhrp2* and *pfhrp3* genes were performed using reaction conditions and primers reported previously [15, 22]. *In vitro* cultured isolate Dd2 was used as a negative control for *pfhrp2* amplification experiments while HB3 served as a negative control for *pfhrp3* amplification because the laboratory isolates lacked the respective genes. The resulting PCR amplicons were visualized on a 2 % agarose gel. If a visible band of the appropriate size was observed, the isolate was scored as positive for the presence of the respective gene and that result was recorded as final. If no amplification occurred, PCR amplification was repeated to confirm this observation. If no amplification occurred during the second amplification attempt, thus showing concordance with the original PCR result, no further testing was done and the outcome was reported as negative. However, if there was discordance between the first and second result, the PCR was repeated again and the two matching results out of three were scored as the final result.

### Microsatellite typing

Genomic DNA was used for microsatellite characterization. Samples were genotyped for seven neutral microsatellite loci spanning six chromosomes that had been used in previous studies (*TA1*, chromosome 6; *Polyα*, ch. 4; *PfPK2*, ch. 12; *TA109*, ch. 6; and *2490,* ch. 10; *C2M34*, ch. 2; *C3M69*, ch. 3) [23–25]. Genomic DNA was amplified by PCR using previously described methods [12, 15, 23–25] and fluorescently labelled (HEX and FAM) PCR products were separated by capillary electrophoresis on an Applied Biosystems 3130xl genetic analyzer (Applied Biosystems Foster City, CA, USA). Alleles were then sized and scored using GeneMapper v3.7 (Applied Biosystems Foster City, CA, USA) and binned to the nearest two or three base pairs.

### Drug resistance markers

Sanger sequencing was performed on all samples to identify drug resistance-associated mutations in *pfcrt, pfdhfr, pfdhps*, and *pfmdr1* genes [26]. Mutations in the codon positions 72–76 of *pfcrt*, 51, 59, 108, and 164 of *pfdhfr*, 436, 437, 540, 581, and 613 of *pfdhps* and 84, 134, 184, 1034, 1042, 1226 and 1246 of *pfmdr1* were identified.

### Network analysis

A median-joining network diagram was generated in Network 4.6.1.3 [27] using the seven neutral microsatellites previously described to examine the genetic relationships among the 32 samples collected from the outbreak, in addition to Ecu 1110, the Peruvian historical isolates published previously [12] as well as five Colombian isolates published recently [14].

### Statistical analysis

Analysis of molecular variance (AMOVA) was used to partition variation within the study parasite population and between the population of Ecuador and falciparum populations of western Peru. In addition, heterozygosity (He) and pair-wise fixation indices (Fst) were calculated. Arlequin 3.5.1.2 software (CMPG, Swiss Institute of Bioinformatics, Lausanne, Switzerland) was used for these analyses [28]. Structure v2.3.4 software [29, 30] was used to assign outbreak samples to previously known populations and Structure Harvester [31] was used to define the number of expected populations.

## Results

During the *P. falciparum* malaria outbreak in Esmeraldas, 151 cases of *P. falciparum* were identified (Fig. 1). Eighty per cent of the cases were located in two parishes in the south part of Esmeraldas. Men accounted for 57 % of the cases. Fifty per cent of the total number of cases were reported in teenagers and young adults (11–25 years old), while 13 % were in children 10 years old or younger, 22 % were in adults 25–40 years old and 15 % were in adults over 40 years old. Most cases were reported between February and April 2013, which corresponds to the rainy season on the Ecuadorian coast (Fig. 1). Thirty-two of these samples were available for this molecular investigation and were confirmed positive by nested PCR and real time PET-PCR.

### Genetic characterization using microsatellite markers

Among the 32 samples tested, 31 showed similar microsatellite profile for all seven microsatellites, as indicated in Table 1. There were some minor variations in four samples. For instance, one locus (TA1) could not be amplified in sample F37 but the rest of the alleles were nearly identical to parasites of the E clonal lineage. Three of the samples (F3, F9 and F26) had extra alleles in locus C3M69 (122 bp) in addition to the shared genotype. With the exception of these minor variations, 31/32 isolates shared a very similar genetic profile corresponding to the E clonal lineage of Peru. One sample (F31) had a distinct genotype from all others, differing at four of seven loci. This isolate was similar to clonal lineage D in at least six of the seven loci tested (Table 1; Additional file 1).

**Table 1:** Microsatellite alleles amplified in isolates collected during the outbreak

Clonal type^a^	Number of samples	TA1 Ch.6	Poly-α Ch.4	PfPK2 Ch.12	TA109 Ch.6	2490 Ch. 10	C2M34 Ch. 2	C3M69 Ch 3.
E	31	171	147	174	160	72	226	140
D	1	171	174	174	160	81	232	122

^a^The clonal types of these parasites were comparable to previously described *P. falciparum* populations in Peru as demonstrated in Additional file 1

**Fig. 4:**
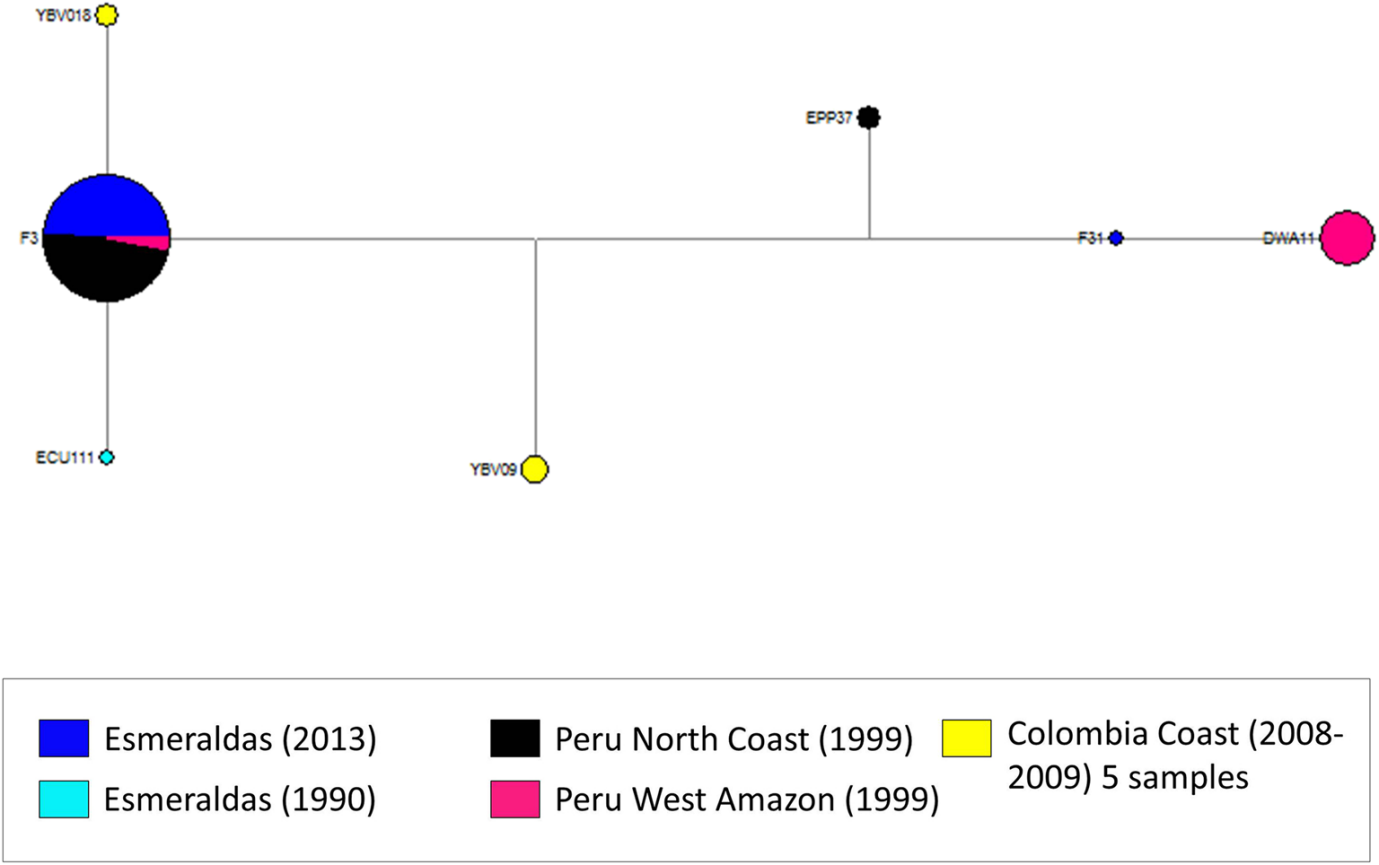
Network analysis of samples in this study and neighboring areas of Peru and Colombia. Network diagram showing genetic relationships among samples from Esmeraldas outbreak (2013), the Ecuadorian clone Ecu 1110 (1990), the coast and west Amazon of Peru (1999) (clonal lineages D and E) as well as five related samples from the Coast of Colombia. The *size of the circles* represent the number of samples. A large majority of samples from the outbreak are closely related to samples from the north coast of Peru, some samples from the west Amazon of Peru and the coast of Colombia. Sample F31 is closely related to clonal lineage D of the West Amazon of Peru (also present in the East Amazon of Peru)

As expected, heterozygosity in the Esmeraldas outbreak samples was very low (He = 0.0625) for most loci, consistent with a majority of isolates being clonal. Pair-wise Fst was performed using the data from seven neutral microsatellite markers and compared with the five clonal lineages previously reported in Peru and four lineages reported in Colombia to determine their genetic relatedness. As shown in Table 2, the Fst value between Peru’s E clonal lineage and the Esmeraldas outbreak population was very close to zero, showing their close genetic relationship (P = 0.505). The pair-wise Fst with the other Peruvian clonal lineages and Colombian lineages were significantly higher (P = 0.00) (Table 2). To confirm the relatedness between the outbreak samples and clonal lineages E and D, a median joining network analysis was performed. The network analysis indicates that most of the samples are linked only to the coastal and Western Amazon parasites of Peru (clonal lineage E) and to the clone Ecu 1110. In addition, five samples from Valle del Cauca in Colombia [14] were closely related to clonal lineage E and most outbreak samples. The exception was sample F31 which appears to be more closely related to clonal lineage D (Fig. 4).

**Table 2:** Pair-wise Fst of Esmeraldas outbreak samples versus Peru samples by clonal lineages [11] and Colombian samples by clusters [15]

Clonal lineage^a^	Sample size	Esmeraldas (Ecu)
Esmeraldas (ECU)	32	
A clonal lineage (PER)	24	0.90963
B clonal lineage (PER)	18	0.94465
C clonal lineage (PER)	26	0.87674
D clonal lineage (PER)	28	0.87538
E clonal lineage (PER)	34	−0.00085
Cluster 1 (COL)	21	0.57805
Cluster 2 (COL)	17	0.70054
Cluster 3 (COL)	26	0.47820
Cluster 4 (COL)	28	0.48890

^a^The neutral microsatellite data reported for clonal lineages A to E (Peru) were obtained from previously published study for this analysis [11]. The neutral microsatellite data reported for Colombian parasites were obtained from previously published studies [14]

### Drug resistance markers

All the samples obtained from the outbreak were tested by sequencing for single nucleotide polymorphisms (SNPs) in the anti-malarial drug resistance genes *pfcrt*, *pfdhfr*, *pfdhps*, and *pfmdr1* (Table 3). All 31 samples that were related to the E clonal lineage carried the *pfcrt* genotype CVMNT (codon 72–76) while the singular D clonal lineage-related isolate, F31, had the CVMET genotype. The E clonal lineage-related 31 samples were wild type for all SNPs tested in the *pfdhfr* and *pfdhps* genes. Sample F31 (D clonal lineage) on the other hand, had the S108 N mutation in *pfdhfr* and a synonymous mutation in codon 540 of *pfdhps*. The Y184F mutation was found in the *pfmdr1* gene of all 31 E clonal lineage parasites while the lone D clonal lineage parasite was wild type at this codon. In addition, all 32 samples had the mutation N1042D in *pfmdr1*. No mutations were found in any of these samples in codons 86, 130, 144, 1034, 1226, and 1246 of *pfmdr1* (Table 3; Additional file 2).

**Table 3:** Drug resistance markers haplotypes and *pfhrp2* and *pfhrp3* presence

	Number of samples	MS clonal type	*Pfcrt*	*Pfdhfr*	*Pfdhps*	*Pfmdr1*	*Pfhrp2*	*Pfhrp3*
Esmeraldas 2013	31 1	E D	CVMN**T** CVM**ET**	CNCSI CNC**N**I	SAKAA SA(**Syn**)AA	NED**F** S**D**FD NEDY S**D**FD	+ −	+ −
Peru clonal lineage E 1999^a^	42	E	CVMN**T**	CNCSI CNC**N**I	SAKAA	NED**F** SNF**Y** NED**F** S**D**F**Y** NEDY SNFD	+	+
Ecu 1110 1990^b^	1	E	CVMN**T**	CNCSI	SAKAA	NED**F** S**D**FD	+	+

*Pfcrt*: 72–76

*Pfdhfr*: 50, 51, 59, 108, 164

*Pfdhps*: 436, 437, 540, 581, 613

*Pfmdr1*: 86,130, 144, 184, 1034, 1042, 1226, 1246

^a^Data as reported in [12]

^b^Data as reported in [34]

### *Pfhrp2* and *pfhrp3* genotyping

The samples from this outbreak were genotyped for the presence of *pfhrp2* and *pfhrp3* genes. The results indicated that both genes were intact in all 31 E clonal lineage-related parasite isolates. In contrast, both *pfhrp2* and *pfhrp3* were deleted in the D clonal lineage isolate F31 (Table 3).

## Discussion

**Fig. 5:**
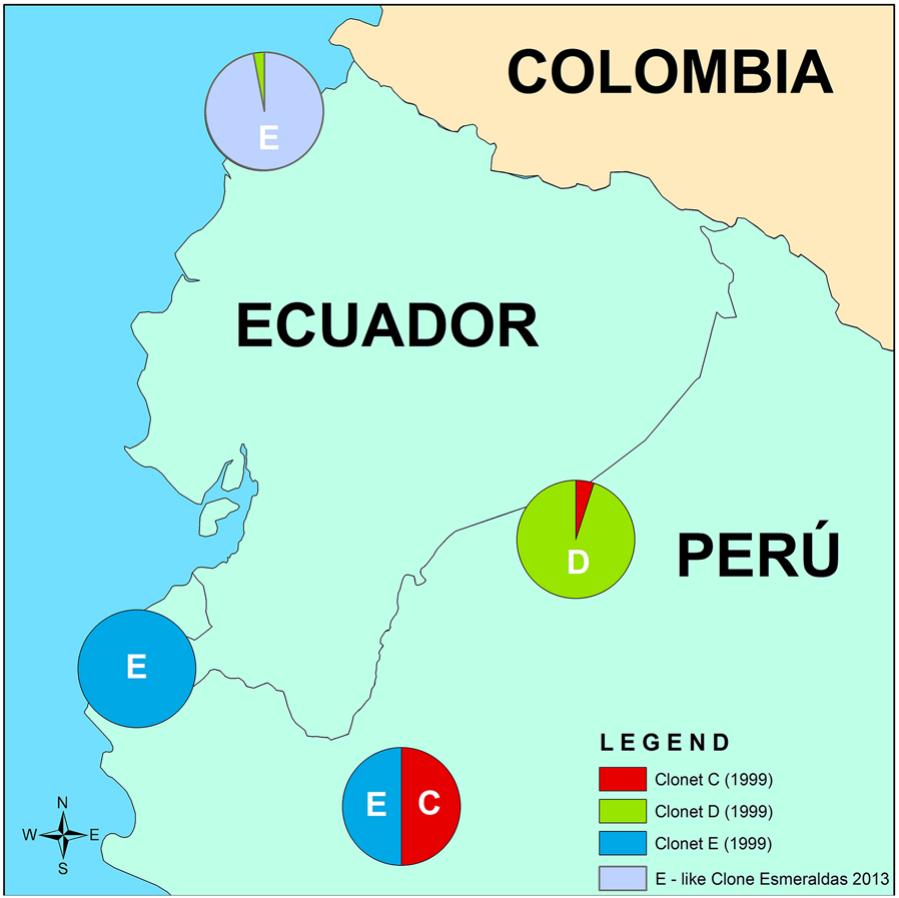
Distribution of clonal lineages in Peru (12) and in Esmeraldas, Ecuador. Esmeraldas outbreak *P. falciparum* parasites from Ecuador share the same microsatellite haplotype as the Peruvian E clonal lineage but differ in some drug resistance markers

As Ecuador progresses towards the pre-elimination phase of the malaria control programme, it is important to thoroughly investigate any malaria occurrences in the country. In this context, understanding the origins of a population of parasites during an outbreak will help the national programme to develop strategies for prevention of future outbreaks. The current molecular epidemiologic investigation has determined that, with the exception of one parasite isolate, the isolates from the 2012–2013 outbreak that were tested carried a nearly identical genotypic profile which is related to the *P. falciparum* E clonal lineage parasites circulating on the north coast of Peru in 1999–2000. Interestingly, a single historical isolate collected in 1990 from the coast of Ecuador was also found to be similar to the E clonal lineage highlighting the historical connection between Peru and Ecuador in sharing a genetically similar population of parasites. These isolates also shared a similar drug resistance marker profile in *pfcrt*, *pfdhfr*, *pfdhps*, and *pfmdr1* and had intact *pfhrp2* and *pfhrp3* genes. Interestingly, a single isolate collected in epidemiological week 38 was found to be distinct from the rest of the outbreak isolates in its genetic profile and was related to another clonal lineage (D) that was reported previously in Peru (Fig. 5).

Overall, these findings suggest that the main source of this outbreak might be parasites belonging to the E clonal lineage. However, not all samples from this outbreak were available due to logistical challenges and the unwillingness of some participants to provide consent. Since not all outbreak samples were available for molecular analysis, it was not possible to identify the genetic profile of all the parasite isolates, which would have helped to unambiguously describe the origin of the entire outbreak parasite population. Nevertheless, this finding clearly illustrates the utility of molecular epidemiological investigations in determining the origin(s) of outbreak parasite populations in future investigations as Ecuador moves towards malaria elimination.

The high clonality of *P. falciparum* observed in this low-transmission area is consistent with reports from Peru [12, 13, 32], Colombia [10, 14, 21] and Panama [5]. Using the same set of microsatellite markers used in this study, recent *P. falciparum* outbreaks in the north coastal region and Cusco area of Peru, were determined to have originated in a region of the Peruvian Amazon [7, 8]. A molecular epidemiological study conducted previously in the north coast of Peru determined that E clonal lineage parasites were circulating in the 1990s [12]. Importantly, *P. falciparum* transmission was eliminated in the north coast of Peru from 2005 until the malaria outbreak of 2010–2012, during which a clonal population of parasites belonging to B_V1_ lineage from Peruvian Amazon, were introduced to the region [8]. These findings demonstrate the utility of molecular tools for epidemiological investigations.

Since the genetic profile of *P. falciparum* parasites that were circulating in Ecuador is not known, it is difficult to clearly attribute whether the current major outbreak population expanded from a residual, vestigial population of parasites that may still be circulating in Ecuador. The finding that a single historical isolate from Ecuador collected about two decades ago genetically resembled the major outbreak population and that sporadic cases have been occurring in the area (Fig. 1) is consistent with the hypothesis that E clonal lineage-related *P. falciparum* strains were present in Ecuador throughout this time period. Based on this finding, one could argue for the possibility of a potential local expansion of residual reservoirs of parasites with E clonal genetic signatures in this outbreak due to some ecological changes. However, further genetic evidence using parasite isolates collected in recent times from this region is needed to support such a hypothesis.

An alternative explanation for the source of this outbreak, based on epidemiological investigations, suggests that the migration of human cases between Colombia and Ecuador may be the cause of this outbreak (Ministry of Public Health of Ecuador, pers. comm.). However, there is no strong evidence to support this hypothesis as explained below. In a recent study, five parasite isolates closely related to the E clonal lineage were detected in sample collections from 2008 and 2009 obtained from Valle de Cauca Department, Colombia. However, three of these parasites differed from this outbreak samples in the chromosomal 10 locus 2490 and two differed in the chromosomal 6 locus TA109. While mostly CVMNK and CVMNT genotypes of *pfcrt* have been reported in Ecuador [33], Colombian parasites have been reported to be CVMET genotype [34]. Since there was no availability of parasite specimens from the index case(s), it was not possible to link the genetic data obtained with travel history information.

The F31 sample donor, who reported travel outside of the Esmeraldas area and worked in the Amazon region of Ecuador (Ministry of Public Health, pers. comm.), had parasites belonging to the D clonal lineage. Interestingly, this parasite isolate carried *pfcrt* genotype of CVMET that is characteristic of Colombian parasites, as documented in previous studies [34, 35]. Based on the travel history and *pfcrt* genotype, one can hypothesize that this traveler may have acquired the D lineage parasites from Colombia or the Ecuador Amazon region; however, information about the genetic profile of *P. falciparum* parasites circulating in the Ecuadorian Amazon is lacking and so in order to validate the hypothesis, *P. falciparum* genotype data will need to be collected from the region.

Ecuador changed its *P. falciparum* anti-malarial treatment policy in 2006, switching from chloroquine (CQ) to artesunate-sulfadoxine-pyrimethamine (AS-SP) and recently to artemether-lumefantrine (AL) [33, 36]. It is important to note that even though the *P. falciparum* parasites from the outbreak have a CQ-resistant haplotype (CVMNT and CVMET), they carried wild type *dhfr* and *dhps* gentotypes associated with SP sensitivity. Mutations in *pfmdr1* were restricted to only two codons as opposed to mutations in three or four codons of this gene in parasite isolates from the Amazon region of South America [13, 37]. Remarkably, the most common haplotype found in the Esmeraldas outbreak had the exact same drug resistance gene profile as Ecu 1110 [34, 35, 38], an isolate collected in Ecuador in 1990. These results suggest that if these outbreak parasites are from the local residual transmission foci then they have maintained the historical CQ-resistant haplotype CVMNT in the population. In addition, the profile of wild type *pfdhr* and *pfdhps* alleles including the limited mutation pattern in *pfmdr1* in the E clonal outbreak parasites indicates that this is an ancestral lineage that may predate SP introduction.

The clonal population from the Esmeraldas outbreak does not have deletions of *pfhrp2* or *pfhrp3*, which would imply that these parasites can be detected by PfHRP2-based rapid diagnostic tests (RDTs). However, the D clonal lineage-related F31 sample had deleted both *pfhrp2* and *pfhrp3*. This finding highlights the need for vigilance when PfHRP2-based RDTs are used for control programmes in South America since *pfhrp2* deletion has been documented in parts of South America [14–16, 18].

## Conclusions

Taken together, this data suggest that the *P. falciparum* isolates from the Esmeraldas outbreak are the result of a clonal expansion of parasites either circulating at very low levels in Ecuador or re-invading Ecuador from border countries. Some of the limitations of this study include the lack of genetic and travel information from the index case(s), lack of population-level genotypic data for *P. falciparum* parasites in Ecuador and the availability of only a sub-set of samples from the outbreak for analysis. Nevertheless, this study highlights the importance of molecular studies to investigate the origin and relatedness of *Plasmodium* parasite populations in outbreak investigations. Further genetic characterization of historical malaria parasites from Ecuador will help in determining if low-level transmission of bottle-necked, vestigial populations of historical Ecuadorian parasites still contribute to local malaria transmission. The genetic characterizations will also help in future outbreak investigations to differentiate residual bottle-necked populations from newly introduced parasites from other regions as efforts are underway to eliminate malaria from this region.

## Additional files

10.1186/s12936-015-1019-2 Microsatellite alleles found in the study samples. Two numbers indicate two alleles detected and DNW indicates no allele amplification at that locus.
10.1186/s12936-015-1019-2 Drug resistance haplotypes and *pfhrp2* and *pfhrp3* presence in the study samples.
