# Supplementary material for: Clonal population expansion in an outbreak of Plasmodium falciparum on the northwest coast of Ecuador
Source: Malar J. 2015 Dec 10;14:497. doi: 10.1186/s12936-015-1019-2 (PMC4676133; doi:10.1186/s12936-015-1019-2)
Supplement: Supplementary file 1 — 10.1186/s12936-015-1019-2 Microsatellite alleles found in the study samples. Two numbers indicate two alleles detected and DNW indicates no allele amplification at that locus. [file 12936_2015_1019_MOESM1_ESM.docx]

**Additional file 1**

**Microsatellite alleles found in the study samples**

Two numbers indicate two alleles detected and DNW indicates no allele amplification at that locus.

| **Chromosome** | **6** | **4** | **12** | **6** | **10** | **2** | **3** |
| --- | --- | --- | --- | --- | --- | --- | --- |
| **Locus** | **TA1** | **Poly-** | **PfPK2** | **TA109** | **2490** | **C2M34** | **C3M69** |
| **Sample ID** | **1** | **3** | **7** | **9** | **12** | **313** | **383** |
| F3 | 171 | 147 | 174 | 160 | 72 | 226 | 122,140 |
| F4 | 171 | 147 | 174 | 160 | 72 | 226 | 140 |
| F6 | 171 | 147 | 174 | 160 | 72 | 226 | 140 |
| F7 | 171 | 147 | 174 | 160 | 72 | 226 | 140 |
| F8 | 171 | 147 | 174 | 160 | 72 | 226 | 140 |
| F9 | 171 | 147 | 174 | 160 | 72 | 226 | 136,140 |
| F10 | 171 | 147 | 174 | 160 | 72 | 226 | 140 |
| F11 | 171 | 147 | 174 | 160 | 72 | 226 | 140 |
| F12 | 171 | 147 | 174 | 160 | 72 | 226 | 140 |
| F13 | 171 | 147 | 174 | 160 | 72 | 226 | 140 |
| F14 | 171 | 147 | 174 | 160 | 72 | 226 | 140 |
| F15 | 171 | 147 | 174 | 160 | 72 | 226 | 140 |
| F16 | 171 | 147 | 174 | 160 | 72 | 226 | 140 |
| F17 | 171 | 147 | 174 | 160 | 72 | 226 | 140 |
| F18 | 171 | 147 | 174 | 160 | 72 | 226 | 140 |
| F19 | 171 | 147 | 174 | 160 | 72 | 226 | 140 |
| F20 | 171 | 147 | 174 | 160 | 72 | 226 | 140 |
| F21 | 171 | 147 | 174 | 160 | 72 | 226 | 140 |
| F22 | 171 | 147 | 174 | 160 | 72 | 226 | 140 |
| F23 | 171 | 147 | 174 | 160 | 72 | 226 | 141 |
| F26 | 171 | 147 | 174 | 160 | 72 | 226 | 122,140 |
| F27 | 171 | 147 | 174 | 160 | 72 | 226 | 140 |
| F28 | 171 | 147 | 174 | 160 | 72 | 226 | 140 |
| F31 | 171 | 174 | 174 | 160 | 81 | 232 | 122 |
| F32 | 171 | 147 | 174 | 160 | 72 | 226 | 140 |
| F33 | 171 | 147 | 174 | 160 | 72 | 226 | 140 |
| F34 | 171 | 147 | 174 | 160 | 72 | 226 | 140 |
| F36 | 171 | 147 | 174 | 160 | 72 | 226 | 140 |
| F37 | DNW | 147 | 174 | 160 | 72 | 226 | 140 |
| F38 | 171 | 147 | 174 | 160 | 72 | 226 | 140 |
| F39 | 171 | 147 | 174 | 160 | 72 | 226 | 140 |
| F40 | 171 | 147 | 174 | 160 | 72 | 226 | 140 |
| E clonet | 171 | 147 | 174 | 160 | 72 | 226 | 140 |
| D clonet | 171 | 159 | 174 | 160 | 81 | 232 | 122 |
| Ecu1110 | 171 | 147 | 174 | 160 | 72 | 232 | 140 |
| 3D7 | 185 | 150 | 168 | 172 | 81 | 260 | 172 |
