# Supplementary material for: Clonal population expansion in an outbreak of Plasmodium falciparum on the northwest coast of Ecuador
Source: Malar J. 2015 Dec 10;14:497. doi: 10.1186/s12936-015-1019-2 (PMC4676133; doi:10.1186/s12936-015-1019-2)
Supplement: Supplementary file 2 — 10.1186/s12936-015-1019-2 Drug resistance haplotypes and pfhrp2 and pfhrp3 presence in the study samples. [file 12936_2015_1019_MOESM2_ESM.docx]

**Additional file 2**

**Drug resistance haplotypes and *Pfhrp2* and *Pfhrp3* presence in the study samples**

| **Haplotype** | ***Pfcrt*** | ***Pfdhfr*** | ***Pfdhps*** | ***Pfmdr1*** | ***Hrp2*** | ***Hrp3*** |
| --- | --- | --- | --- | --- | --- | --- |
| **Sample ID** |  |  |  |  |  |  |
| F3 | CVMNT | CNCSI | SAKAA | NEDFSDFD | + | + |
| F4 | CVMNT | CNCSI | SAKAA | NEDFSDFD | + | + |
| F6 | CVMNT | CNCSI | SAKAA | NEDFSDFD | + | + |
| F7 | CVMNT | CNCSI | SAKAA | NEDFSDFD | + | + |
| F8 | CVMNT | CNCSI | SAKAA | NEDFSDFD | + | + |
| F9 | CVMNT | CNCSI | SAKAA | NEDFSDFD | + | + |
| F10 | CVMNT | CNCSI | SAKAA | NEDFSDFD | + | + |
| F11 | CVMNT | CNCSI | SAKAA | NEDFSDFD | + | + |
| F12 | CVMNT | CNCSI | SAKAA | NEDFSDFD | + | + |
| F13 | CVMNT | CNCSI | SAKAA | NEDFSDFD | + | + |
| F14 | CVMNT | CNCSI | SAKAA | NEDFSDFD | + | + |
| F15 | CVMNT | CNCSI | SAKAA | NEDFSDFD | + | + |
| F16 | CVMNT | CNCSI | SAKAA | NEDFSDFD | + | + |
| F17 | CVMNT | CNCSI | SAKAA | NEDFSDFD | + | + |
| F18 | CVMNT | CNCSI | SAKAA | NEDFSDFD | + | + |
| F19 | CVMNT | CNCSI | SAKAA | NEDFSDFD | + | + |
| F20 | CVMNT | CNCSI | SAKAA | NEDFSDFD | + | + |
| F21 | CVMNT | CNCSI | SAKAA | NEDFSDFD | + | + |
| F22 | CVMNT | CNCSI | SAKAA | NEDFSDFD | + | + |
| F23 | CVMNT | CNCSI | SAKAA | NEDFSDFD | + | + |
| F26 | CVMNT | CNCSI | SAKAA | NEDFSDFD | + | + |
| F27 | CVMNT | CNCSI | SAKAA | NEDFSDFD | + | + |
| F28 | CVMNT | CNCSI | SAKAA | NEDFSDFD | + | + |
| F31 | CVMET | CNCNI | SA(syn)AA | NEDYSDFD | - | - |
| F32 | CVMNT | CNCSI | SAKAA | NEDFSDFD | + | + |
| F33 | CVMNT | CNCSI | SAKAA | NEDFSDFD | + | + |
| F34 | CVMNT | CNCSI | SAKAA | NEDFSDFD | + | + |
| F36 | CVMNT | CNCSI | SAKAA | NEDFSDFD | + | + |
| F37 | CVMNT | CNCSI | SAKAA | NEDFSDFD | + | + |
| F38 | CVMNT | CNCSI | SAKAA | NEDFSDFD | + | + |
| F39 | CVMNT | CNCSI | SAKAA | NEDFSDFD | + | + |
| F40 | CVMNT | CNCSI | SAKAA | NEDFSDFD | + | + |
| E clonet^#^ | CVMNT | CNCSI | SAKAA | NEDFSDFD | + | + |
| D clonet^#^ | CVMET | CNCNI | SA(syn)AA | NEDYSDFD | - | - |
| Ecu1110* | CVMNT | CNCSI | SAKAA | NEDFSDFD | + | + |
| 3D7 | CVMNK | CNCSI | SGKAA | NEDYSNFD | + | + |

*Pfcrt*: 72-76

*Pfdhfr*: 50, 51, 59, 108, 164

*Pfdhps*: 436, 437, 540, 581, 613

*Pfmdr1*: 86,130, 144, 184, 1034, 1042, 1226, 1246

# Data as reported in [11]

* Data as reported in [32]
